# Risk of Deficiency in Multiple Concurrent Micronutrients in Children and Adults in the United States

**DOI:** 10.3390/nu9070655

**Published:** 2017-06-24

**Authors:** Julia K. Bird, Rachel A. Murphy, Eric D. Ciappio, Michael I. McBurney

**Affiliations:** 1 Nutrition Innovation Center, Human Nutrition and Health, DSM Nutritional Products, Kaiseraugst CH-4303, Switzerland; 2 School of Population and Public Health, University of British Columbia, Vancouver, BC V6T 1Z3, Canada; rachel.murphy@ubc.ca; 3 Scientific Affairs, DSM Nutritional Products, Parsippany, NJ 07054, USA; eric.ciappio@dsm.com (E.D.C.); michael.mcburney@dsm.com (M.I.M.)

**Keywords:** NHANES, nutritional status, deficiency, dietary adequacy, nutritional epidemiology, dietary supplement, multivitamin-mineral

## Abstract

Certain population sub-groups in the United States are vulnerable to micronutrient malnutrition. Nationally representative data from the National Health and Nutrition Examination Survey (NHANES) describing the biochemical status of vitamins A, B6, B12, C, D, E, folate, and anemia, were aggregated to determine the overall risk of multiple concurrent deficiencies in U.S. children and adults (*n* = 15,030) aged >9 years. The prevalence of deficiency risk according to socio-demographic, life-stage, dietary supplement use, and dietary adequacy categories was investigated. Thirty-one percent of the U.S. population was at risk of at least one vitamin deficiency or anemia, with 23%, 6.3%, and 1.7% of the U.S. population at risk of deficiency in 1, 2, or 3–5 vitamins or anemia, respectively. A significantly higher deficiency risk was seen in women (37%), non-Hispanic blacks (55%), individuals from low income households (40%), or without a high school diploma (42%), and underweight (42%) or obese individuals (39%). A deficiency risk was most common in women 19–50 years (41%), and pregnant or breastfeeding women (47%). Dietary supplement non-users had the highest risk of any deficiency (40%), compared to users of full-spectrum multivitamin-multimineral supplements (14%) and other dietary supplement users (28%). Individuals consuming an adequate diet based on the Estimated Average Requirement had a lower risk of any deficiency (16%) than those with an inadequate diet (57%). Nearly one-third of the U.S. population is at risk of deficiency in at least one vitamin, or has anemia.

## 1. Introduction

Numerous sources including the 2015 Dietary Guidelines Advisory Committee Report have highlighted shortfalls in key nutrients within the U.S. population [1,2]. Adequate intakes of micronutrients are essential for supporting the growth and development of children, as well as maintaining overall health across the lifespan. A prolonged, inadequate intake of essential micronutrients results in deficiencies that negatively impact health. Deficiency symptoms include impaired immunity, growth and night blindness from vitamin A deficiency [3], impaired wound healing and bleeding from vitamin C deficiency [4], anemia from iron deficiency [5], and rickets and osteomalacia from vitamin D deficiency [6]. Deficiencies in the B vitamins lead to different types of anemia: folate deficiency leads to megaloblastic anemia, vitamin B6 deficiency results in microcytic anemia, whereas vitamin B12 deficiency causes pernicious anemia, and may result in neurological damage due to impaired myelination [7]. An adequate status of micronutrients in combination is required for many important processes in the body. For example, erythropoiesis requires not only iron, but also folate, vitamin B12, and vitamin A, and dietary vitamin C can improve the absorption of non-heme iron [8]. Sub-clinical deficiency symptoms for many vitamins and minerals are non-specific, and may include fatigue, irritability, aches and pains, decreased immune function, and heart palpitations [4,7].

While estimates of the vitamin and mineral status of the U.S. population have been undertaken for many decades, the initiation of the National Health and Nutrition Examination (NHANES) surveys in the 1970s greatly improved access to representative data on nutrient intakes and deficiencies [9,10]. Cohort studies such as the Framingham Heart Study [11] and the Multiethnic Cohort [12], and large clinical trials [13,14] also provided valuable insights into vitamin intakes and status in certain population groups. The current estimates indicate that vitamin A, vitamin D, vitamin E, folate, vitamin C, calcium, and magnesium are under-consumed relative to the Estimated Average Requirement (EAR), while iron is under-consumed by adolescent and adult females, including those who are pregnant [1]. The Centers for Disease Control and Prevention (CDC) measured biochemical indicators of diet and nutrition in a representative sample of the U.S. population from 2003–2006 [15], and a series publications explores this data in more detail [2,16,17,18,19,20]. From the main report, the deficiency prevalence for each of the vitamins B6, C, and D, and the mineral iron, ranged between 5–10%. The deficiency prevalence estimates were investigated according to ethnicity, age, and gender and showed that women and non-Hispanic Blacks tended to have greater vitamin B6 deficiency, older adults and non-Hispanic Whites had a greater prevalence of vitamin B12 deficiency, men and non-Hispanic Whites had a greater prevalence of vitamin C deficiency, and almost one in three non-Hispanic Blacks had a vitamin D deficiency. However, the report does not examine the prevalence rates in risk groups such as pregnant and breastfeeding women, low-income households, or according to educational status, body mass index (BMI), or measures of dietary intake. A general estimate of the prevalence of multiple concurrent deficiencies was conducted, including a sub-group analysis in a single risk group [2]. This analysis found that although 78% of the U.S. population aged over 6 years was not at risk of deficiency, only 74% of women of childbearing potential (aged 12–49) were not at risk of deficiency, and when iron-deficiency anemia was included, 68% were not at risk of deficiency. The analysis also found that 5.7% of the U.S. population was at risk of two or more vitamin deficiencies. Despite considerable interest in deficiency in single vitamins or minerals, we are not aware of any other estimates of aggregated vitamin or mineral deficiencies in the U.S. population, although some smaller surveys have measured the biochemical status of multiple micronutrients in risk populations in other countries [21,22,23,24].

Dietary supplements (DS) can be an important source of vitamins and minerals to prevent inadequate dietary intakes. Approximately half of adults and one third of children report DS use [25,26,27], primarily in the form of multivitamins with or without minerals. DS users have a lower prevalence of inadequate micronutrient intake among adults [28,29,30], children, and adolescents [29,31]. In the U.S., DS are used most often to maintain or improve overall health [25,32]. DS are widely used as “nutritional insurance” to cover unintended gaps in dietary intakes [25,33].

Although a commonly used definition of a multivitamin-multimineral supplement is that it contains at least three vitamins and at least one mineral [26], this definition is very broad and captures not only DS intended to be taken every day to fill dietary gaps, but also specialized formulations targeted at specific health benefits such as eye or bone health, or sports supplements. We wanted to investigate whether there was any difference in rates of deficiency when individuals used “full spectrum” multivitamin-multimineral DS (FSMV), which contain all 12 vitamins and the most nutritionally important minerals, i.e., calcium, iron, iodine, magnesium, zinc, selenium, copper, manganese, chromium, and molybdenum. These types of supplements have been used in several long-term clinical trials [34,35,36], and align more closely with consumer use.

The aims of this study are (1) to determine the risk of deficiency for multiple micronutrients in the U.S., (2) to identify groups with a greater burden of deficiency risk across a broad range of socio-demographic and life span groups, and (3) to determine whether the risk of deficiency differs between DS non-users, DS users, and FSMV users, in the context of dietary adequacy.

## 2. Materials and Methods

### 2.1. Description of Dataset

The National Health and Nutrition Examination Survey (NHANES) is a representative survey of the civilian, non-institutionalized U.S. population, and is designed to assess general health and nutritional status. The National Center for Health Statistics Research Ethics Review Board reviewed and approved protocol #98-12 for data collected in the 2003–2004 cycle, and protocol #2005-06 for data collected in the 2005–2006 cycle. NHANES protocols receive ethical review annually, and ongoing changes are submitted through an amendment process [37]. All of the subjects gave their informed consent for inclusion before they participated in the study. The datasets are publicly available from the National Center for Health Statistics. A complex, multistage probability sampling design is used to select a sample representative of the U.S. population, with a subset of participants undergoing biochemical assessments [38].

For our research, we conducted a secondary analysis of 15,030 participants aged 9 years and over for which demographic data were available from the 2003–2004 and 2005–2006 data cycles. These survey years were chosen as they provide the most comprehensive and recent data describing biochemical nutrient status for multiple vitamins and minerals. We included socio-demographic data for age, sex, race/ethnicity, poverty income ratio (PIR, a measure of household income relative to household size), and educational status. Race/ethnicity was used as defined in NHANES (Non-Hispanic White, Non-Hispanic Black, Mexican American; the results for the “Other Hispanic” and “Other Race” categories are not reported due to small sample size). The PIR was categorized as low (<1.85), medium (≥1.85 and <3.5), or high (≥3.5) [18]. Education for adults aged 20 y and over was categorized as “less than high school”, “high school graduate”, and “some college, or college graduate”. The BMI was calculated from height and weight measured during the medical examination, and was categorized as underweight, normal weight, overweight, or obese according to the standard cut-off points for adults aged 20 years and over [18]. Pregnancy status was determined either by self-report or a laboratory test taken during the physical examination. Self-reported current breastfeeding in women 1 year postpartum or less in the reproductive health questionnaire was used to ascertain breastfeeding status. Age, sex, pregnancy status, and breastfeeding status were used to assign participants to Dietary Reference Intake (DRI) categories used by the Institute of Medicine [7].

### 2.2. Criteria for Determining Biochemical Vitamin and Mineral Status, Biochemical Deficiency Score and Dietary Inadequacy Score

The 2003–2006 NHANES cycles are unique in that they provide comprehensive biochemical measures of micronutrient status for vitamins A, B6, B12, C, D, E, folate, and iron. We used the cut-off points as summarized in Table 1 to identify individuals with biomarker concentrations at risk of deficiency [2]. The cut-offs from the CDC report on biomarkers of nutrient status were used to ascertain the risk of deficiency for vitamins A, B6, B12, folate, C, D, and E [15]. Because the vitamin B6 analysis changed between the 2003–2004 cycle (enzymatic assay) and the 2005–2006 cycle (HPLC method) [39], the data from each cycle are analyzed and reported separately. Vitamin B12 deficiency was defined as either a low serum vitamin B12 (<200 pg/mL) or elevated methylmalonic acid ((MMA); >0.271 µmol/L) [15]. Similarly to the CDC report, any participant with either a low serum folate (<2 ng/mL) or low red blood cell folate (<95 ng/mL) was defined as deficient in folate [15]. Iron deficiency anemia is best determined using a combination of serum ferritin to describe low iron stores, and hemoglobin to determine anemia [40]. However, the serum ferritin test is only available for a limited population within NHANES, namely women of reproductive age and children aged from 1 to 5 years. To assess the entire population, the iron deficiency anemia screening criteria used by the Association of American Family Physicians were used, which uses a combination of low hemoglobin concentrations and a small mean corpuscular volume to detect individuals with anemia who are at risk of iron deficiency anemia [41].

In NHANES, the dietary intake of vitamins and minerals is estimated by two 24 h dietary recalls conducted on non-consecutive days. The mean of two 24 h dietary recalls was used to estimate dietary inadequacy, insufficiency, and excess based on the EAR, Recommended Dietary Allowance (RDA), and Tolerable Upper Limit (TUL), respectively. For each individual, binary categories were created for nutrient status and nutrient intake; a 1 was assigned if the subject’s biomarker of nutrient status indicated a risk of deficiency (status), and a 1 when an individual failed to meet his/her age-gender and lifespan specific EAR or RDA for a nutrient, or was above the TUL (intake). When the status or intake for a nutrient was sufficient or adequate, a 0 was assigned. Only subjects with complete data for all biomarkers of nutrient status were used when calculating proportions deficient for multiple deficiencies; other subjects were coded as missing for the summed variable. For the biochemical markers of nutrient status, participants were given a score of 0 to 5 based on the number of vitamins or minerals for which they were below the cut-off for deficiency (no participant was at risk of deficiency for more than five vitamins, or had anemia), and a dietary inadequacy/insufficiency score of 0 to 7 based on the number of micronutrients for which their dietary intake was inadequate/insufficient (Supplementary Tables S1 and S2). Scores describing the risk of multiple deficiencies were aggregated to avoid small cell sizes. Groups were defined as: no deficiency; risk of deficiency in one vitamin, or anemia; risk of deficiency in two vitamins, or anemia; risk of deficiency in three to five vitamins, or anemia for 2-way tables. For 3-way tables, we categorized subjects as either no deficiency; or risk of deficiency in one to five vitamins, or with anemia.

### 2.3. Selection of Full Spectrum Multivitamin-Multimineral Supplements

The DS used in NHANES 2003–2006 were categorized according to the count of vitamins and minerals in each product. The FSMV category was based on a large cluster of products with a composition that included a broad range of micronutrients, and thus was defined as users of any DS containing ≥12 vitamins and 7 to 16 minerals, as shown in Supplementary Table S3. Based on this definition, participants were classified as DS non-users, FSMV users, and DS users.

### 2.4. Statistical Methods

All analyses were performed using SAS version 9.3 (SAS Institute Inc., Cary, NC, USA). Statistical significance was set at 0.05, and adjusted by the Bonferroni correction for multiple tests for all sub-group analyses. Procedures that take into account the complex survey design of NHANES were used to produce means and percentages. The Mobile Examination Center sample weight provided by the CDC, adjusted for the 2003–2006 NHANES cycles, was used to create nationally representative estimates for those analyses that did not include dietary analysis, and the Day 2 Dietary sample weights were used for analyses that used the dietary intake datasets. Differences between categorical variables were assessed by comparing confidence intervals with an alpha adjusted by the Bonferroni correction for the number of sub-groups. Significant differences within multiple sub-group categories were marked with superscripts generated according to the method of Dallal [42]. Estimates with a relative standard error greater than 30% were flagged because they lack sufficient precision, as recommended by the CDC [43]. Confidence intervals for proportions were calculated using the SURVEYFREQ procedure, which by default produces Wald (linear) confidence intervals. For confidence intervals of extreme proportions (in our dataset, any confidence interval that included zero as the lower bound), Clopper–Pearson (exact) confidence intervals were computed, as marked in the tables, per the analytical guidelines [43].

## 3. Results

### 3.1. Individual Biochemical Deficiencies and Insufficient Intakes of Vitamins and Minerals

The most common biochemical deficiency in the U.S. population aged ≥9 years was vitamin B6 (Table 1). The proportion of participants at risk of vitamin B6 deficiency was 20% for the 2003–2004 cycle and 11% for the 2005–2006 cycle. A risk of deficiency in vitamins B12, C, and D was found in 5.0%, 6.2%, and 8.9% of the U.S. population, respectively. Anemia was found in 4.3% of the U.S. population overall. Less than 1% of the population was at risk of deficiency for vitamin A, folate, or vitamin E. Demographic characteristics of the study population and biochemical status are already well described in the literature [15,16,29], therefore these data are presented in Supplementary Tables S4 and S5, respectively.

### 3.2. Overall Inadequate Biochemical Status According to Demographic Characteristics

The prevalence of deficiency risk in multiple, concurrent vitamins, or anemia, are reported in Table 2. Sixty-nine percent was not at risk of deficiency, and 23%, 6.3%, 1.5%, 0.14%, and 0.053% was at risk of deficiency in one, two, three, four and five vitamins or had anemia, respectively. The prevalence of deficiency risk or anemia was higher in NHANES cycle 2003–2004 than 2005–2006 due to differences in the analytical method used for vitamin B6. Across all demographic, age, and gender groups, a risk of deficiency in a single nutrient or anemia was observed more frequently than multiple concurrent nutrient deficiencies.

The risk of deficiency in 1, 2, or 3–5 vitamins or anemia was higher among females than males (*p* < 0.0125). There were significant differences in deficiency risk by ethnicity: non-Hispanic Whites had the lowest risk whereas non-Hispanic Blacks had the highest risk of deficiency or anemia (*p* < 0.0125). Individuals from low PIR households were more likely to be at risk of deficiency/anemia, compared to the two higher household income categories (*p* < 0.0125). Completing some college or having a college diploma was associated with a lower risk of deficiency in one or two vitamins/anemia compared to participants without a high school diploma (*p* < 0.0125), although there was no significant difference found according to educational attainment for individuals deficient in three or more vitamins.

There was a U-shaped relationship when the deficiency risk was investigated according to BMI. Both underweight and obese individuals had an increased risk of deficiency compared to normal weight and overweight subjects (*p* < 0.0125). Overall, women who were pregnant had a non-significant higher risk of vitamin deficiency or anemia than women of childbearing potential (defined in the NHANES survey as girls aged 8–11 years who had started menstruating, and all girls and women aged 12–59 years who were not pregnant). On the other hand, the extra nutritional demands of lactation did not appear to result in a higher risk of vitamin deficiency or anemia in postpartum women reporting breastfeeding.

### 3.3. Biochemical Deficiencies across Age and Gender Categories

The overall risk of vitamin deficiency or anemia was investigated according to age, gender, and life stage groups in Table 3. A risk of deficiency/anemia was most frequent in pregnant or breastfeeding females, females aged 19–50 years, and adolescent females 14–18 years. The pattern of low status in certain micronutrients varied by age and gender groups (Table 4), although no significant differences were found for vitamin A and folate (*p* < 0.00625). Male adolescents aged 14–18 years had higher rates of low vitamin E (3.7%) status. Males aged 19–50 years had higher rates of biochemical vitamin C deficiency (8.7%). Females aged 19–50 years were more likely to have a deficient vitamin D status (12%). Vitamin B12 deficiency rates tended to increase with age, with higher rates found in adults aged 51–70 years (6.9%) and 71 years and over (15%) than some younger age groups. The oldest age group (adults 71 years and older) was also more likely to be deficient in vitamin D (9.1%) and have anemia (8.9%). Pregnant and breastfeeding women were more likely to have a deficient status of vitamin B6 (35%) or be anemic (18%). The difference in deficiency prevalence between the 2003–2004 and 2005–2006 vitamin B6 samples appeared to be affected by the age of the subjects and was much more apparent in younger age groups in 2003–2004, and in older age groups in 2005–2006 (results not shown).

### 3.4. Risk of Vitamin Deficiency or Anemia by Dietary Supplement Use Categories and Age/Gender Groups

Younger age groups reported less frequent use of DS and FSMV than older age groups, and women reported greater use than men (Table 5). Pregnant or breastfeeding women were more likely to use DS than non-pregnant women aged 19–50 years, although the use of FSMV was similar.

DS non-users had the highest risk of deficiency (40%) compared to DS users (28%), whereas users of FSMV had the lowest risk of deficiency (14%, *p* < 0.0167). Similar trends were found in all DRI groups, although the differences did not always reach statistical significance.

### 3.5. Risk of Vitamin Deficiency According to Dietary Sufficiency Score and Dietary Supplement Use

A low proportion of the U.S. population has an adequate diet. Based on the EAR, 6.4% of the population consumed at least the EAR for each of the vitamins A, B6, B12, C, E, folate, and iron (Supplementary Table S2). Most people did not meet the EAR for vitamin E (89% inadequate), and approximately half the population did not meet the EAR for vitamins A (52% inadequate) and C (48% inadequate; Supplementary Table S1).

**Figure 1:**
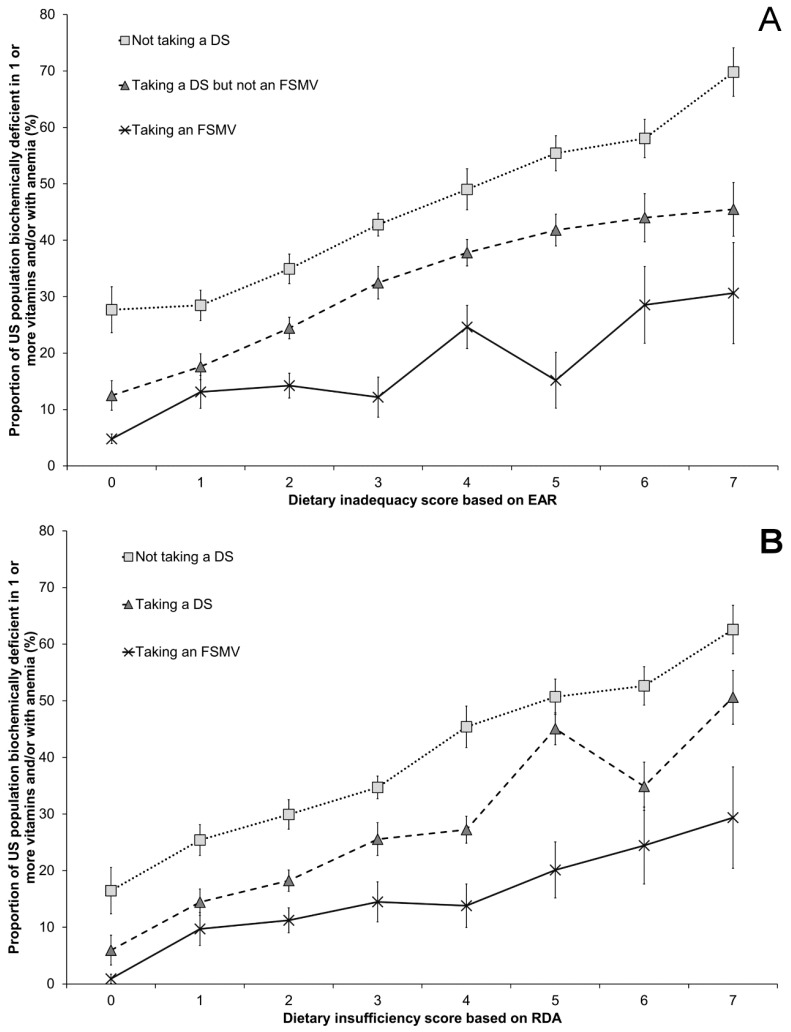
Risk of biochemical vitamin deficiency or anemia, by DS use and dietary vitamin/mineral inadequacy/insufficiency. Dietary inadequacy score reflects the combined number of vitamins and/or minerals for which intake is below the (**A**) Estimated Average Requirement (EAR) or (**B**) Recommended Dietary Allowance (RDA) for vitamins A, B6, B12, C, E, folate, and the mineral iron. Proportions are a percentage of the US population at risk of deficiency in one or more vitamin or with anemia according to biochemical measurements of nutritional status, and error bars reflect the SE. NHANES, National Health and Nutrition Examination Survey; DS, dietary supplement; FSMV, full-spectrum multivitamin-multimineral supplement containing 12 or more vitamins and 7 to 16 minerals.

A lower dietary inadequacy/insufficiency score and reported DS use were both associated with a lower risk of nutrient deficiencies (Figure 1). For subjects who met their requirements for all of the vitamins and minerals in our analysis (dietary inadequacy score of 0 based on the EAR), 28% of DS non-users, 12% of DS users, and 4.8% of FSMV users were at risk of at least one biochemical deficiency. For subjects with a diet that was highly likely to meet their individual requirements (dietary insufficiency score of 0 based on the RDA), 16% of DS non-users, 6.0% of DS users, and 0.9% of FSMV users were at risk of deficiency for one vitamin or mineral. As the number of dietary inadequacies or insufficiencies increased, the risk of deficiency also increased. In subjects with the poorest diets based on the EAR (dietary inadequacy score of 7), the deficiency risk was 70% in DS non-users, 45% in DS users, and 31% in FSMV users. Based on a dietary insufficiency score derived from the RDA (dietary insufficiency score of 7), the risk of deficiency was 63% in DS non-users, 51% in DS users, and 29% in FSMV users. The proportion of the population with intakes above the Tolerable Upper Limit was low: 1.1%, 0.68%, and 0.31% of the population had excessive intakes of iron, folate, and retinol, respectively, and excessive intakes were not found for the other vitamins (Supplementary Table S2). A sensitivity analysis was conducted to determine whether the length of time taking a dietary supplement, or frequency of taking a dietary supplement over the previous 30 days, had an effect on the deficiency score. While there were general trends towards a lower risk of deficiency when dietary supplements were taken more often in the past 30 days, or for longer than 2 months, the results were not statistically significant (Supplementary Tables S6 and S7).

## 4. Discussion

Our analysis showed that nearly one third of the U.S. population aged over 9 years is at risk of deficiency in at least one vitamin, or has anemia. Vulnerable groups include females, especially pregnant or breastfeeding females, non-Hispanic Blacks, participants with a low socio-economic status, and underweight and obese individuals. While DS users had a moderately lower risk of deficiency compared to non-users, users of FSMV, in particular, had a much lower deficiency risk. Individuals consuming their EAR or RDA were less likely to be at risk of deficiency, a relationship that was consistent within DS use categories.

Our deficiency risk data for individual vitamins and minerals agree with other NHANES analyses [4,15,16,44,45,46,47,48,49]. The investigation of multiple concurrent deficiencies conducted using the 2005–2006 cycle of data by Pfeiffer et al. found a lower prevalence of deficiency than our analysis due to differences in rates of deficiencies between survey cycles, our exclusion of young children from the analysis due to uncertainty over appropriate biochemical cut-off values, and differences in the lowest age of children for whom biochemical status was determined between micronutrients. As discussed in more detail below, our use of indicators of iron status for the entire population also had a small impact on the deficiency risk. Our vitamin B6 deficiency estimates agree with Morris et al. [49] for the 2003–2004 data cycle, and with the CDC for the 2005–2006 data cycle [15]. The differences in the prevalence of vitamin B6 deficiency between the 2003–2004 and 2005–2006 cycles reflect changes in analytical methodology.

The CDC reports iron deficiency anemia prevalence based on criteria (hemoglobin and serum ferritin concentrations) that were only available for a limited subset of the population. As we were interested in estimating iron deficiency anemia in the general population, we could only use the less specific blood hemoglobin and mean cell volume measurements. Cogswell et al. provide estimates of the specificity of anemia concentrations to predict iron deficiency anemia in the same survey years as our analysis [50]. In the Cogswell analysis, 6.2% of non-pregnant women aged 12–49 years had anemia, and of this percentage, 76% had iron deficiency anemia. In this population that is at greatest risk of iron deficiency anemia, using the hemoglobin measurement alone results in an overestimation of the prevalence of iron deficiency anemia. It is difficult to predict whether a similar proportion of participants with anemia in other life stage groups have iron deficiency anemia, however it is reasonable to assume a similar ratio. Therefore, our estimations of the prevalence of anemia in the U.S. population are greater than the prevalence of iron deficiency anemia, and it is likely that the actual prevalence of iron deficiency anemia is approximately three quarters of our estimate of 4.3% with anemia.

The finding that women of childbearing age have an elevated risk of vitamin or mineral deficiency has been reported by others [51,52]. In pregnant women, plasma volume expansion may dilute the blood biomarkers of nutrient status, leading to an apparent increase in the risk of deficiency [53,54]. The medical and nutritional significance of plasma volume expansion on markers of nutrient status is unknown. The trend to lower rates of biochemical deficiency in women who breastfeed may relate to higher socio-economic status or better knowledge of nutrition [55]. The markedly higher rates of vitamin D deficiency in non-Hispanic Blacks, found to be 31% in the U.S. population aged 1 year and older for the same survey years and vitamin D cut-off by the CDC [15], is likely to be primarily responsible for the overall greater risk of any deficiency in this racial/ethnic group. In addition, others have found that non-Hispanic blacks are at greater nutritional risk, and this may be due to poorer diets or nutrient intakes [46,56,57]. Inadequate diets found in individuals from lower socio-economic status households [58] may be the cause behind their increased risk of deficiency. Metabolic disturbances related to obesity could be both a cause and a consequence of vitamin deficiency, particularly for vitamins C, B12, folate, and the fat-soluble vitamins, explaining our finding that there was an increased risk of deficiency in obese participants [59].

Given that dietary supplements improve nutrient intakes in general [29], the observation that DS use is associated with a reduced risk of deficiency is consistent with previous observations in the general population, such as has been shown for vitamins B6 [49], B12 [44], and C [4], as well as for folate in pregnant women [45], and for an FSMV in older adults [60]. DS users tend to have a better diet than non-users [31,32,61,62,63], therefore improvements in nutritional status may be the result of a more nutritious diet rather than DS use. We considered DS use within the context of dietary adequacy/sufficiency and found that a small proportion of participants consuming a diet that met the EAR or RDA still were at risk of deficiency. DS use was associated with a lower risk of deficiency even in well-nourished individuals. FSMV users have a lower risk of deficiency than other DS users, therefore it appears that the type of DS used is important. Individuals who take DS containing a wide range of micronutrients have a lower risk of deficiency, irrespective of the adequacy of their diet.

The strengths of our analysis are that the NHANES dataset is large and well-defined, and therefore can provide a robust cross-sectional analysis of the nutrient status of the U.S. population. Our analysis provides insights into nutrient status across a life cycle, including children, adolescents, and pregnant or breastfeeding women. These population groups are nutritionally vulnerable due to higher demands, and data on their nutritional status and needs should be a priority. We attempted to consider the biochemical markers of nutrient status together with measures of dietary sufficiency.

Nevertheless, our research has some limitations. For pregnant and breastfeeding women, in particular, the sample size is small and may not be nationally representative. The biochemical markers were assessed at a single time point, dietary intakes were based on two 24 h dietary recalls conducted in the period around the time of blood collection, and supplement use was measured over the 30 days prior to the dietary interview. There are uncertainties in using a single biochemical measurement to determine micronutrient status, which should ideally be diagnosed after a physical examination, and a dietary history can be taken to place the laboratory results in perspective. When the “index of individuality”—a measure of the variability of individuals’ biochemical measures compared to the population reference interval—is low (<0.6), as it is for folate and vitamins A, B12, and E, our approach is limited to assessing current nutrient status in individuals since it would not be robust enough to detect changes of disease status [64]. Similar analyses of biological variation are lacking for vitamins D, C, and B6.

The biomarkers of nutrient status are affected by inflammation, and this may have affected our results. In the survey years that we analyzed, the vitamins and minerals significantly affected by inflammation (defined as elevated C-reactive protein ≥5 mg/L) were serum and red blood cell (RBC) folate, PLP, and vitamins C, D, and E [16]. Depending on the sub-groups’ level of inflammation, and the effect of inflammation on each biomarker, the deficiency prevalence in certain sub-groups could be affected [65]. For example, serum PLP is strongly depressed when C-reactive protein is elevated, which could mean that the risk of deficiency is lower than estimated in sub-groups more affected by inflammation, such as women, current smokers, or non-Hispanic Blacks [16,65]. Nevertheless, the application of standard cut-off points provides a snapshot of the prevalence of micronutrient deficiencies among individuals in a nationally representative survey, and represents standard clinical practice in identifying micronutrient deficiencies.

FSMV were defined by the number of vitamins and minerals contained within each product, and not their vitamin or mineral content. It is therefore possible that some FSMV products do not contain all of the vitamins and/or minerals for which biochemical status was assessed. Moreover, supplements may not have been used daily. Even so, as they contained at least 12 vitamins, it is unlikely that there are DS within the FSMV category that do not contain all the vitamins for which biochemical data were available. We limited the number of minerals in the supplement to avoid including unusual formulations containing rare earth metals, and it is possible that some FMSV formulations did not contain iron.

Biochemical deficiency did not correlate well with the dietary intake data for each vitamin. This discrepancy may reflect recall bias known to be a problem with dietary intake methodologies [66]. A further issue relates to the use of a limited number of dietary recall days to assess usual intakes, because they are influenced by the day-to-day variation of the diet and will not accurately reflect long-term usual intakes [5]. Differences seen may also reflect changes in the distribution of nutrient intakes related to biochemical status. This could be related to macro-level shifts in micronutrient sources in a diet, or inherent difficulties in applying the results of small-scale micronutrient depletion–repletion studies used to establish DRIs to heterogeneous populations. Nevertheless, biochemical deficiency rates increased as dietary inadequacy/insufficiency increased, an association that was also consistent in sub-group analyses, lending weight to our findings.

Deficiencies and dietary inadequacies in vitamins and minerals have been found in other well-nourished populations. For example, a low riboflavin status was found in 57% of young UK women [67], intakes were below the estimated average requirement for thiamine (26%), zinc (39%), vitamins B6 (25%), and vitamin B12 (27%) in young Canadian women with mood disorders [68], 17% of New Zealand women in Dunedin had a zinc status indicative of mild deficiency [69], and magnesium status in 18% of Canadian women of South-Asian background was low [70]. Regarding international surveys, intakes of iodine, magnesium, iron, and vitamin D were considered to be a concern in a review of several European countries [71], and over 30% of community-dwelling older adults had intakes of vitamin D, thiamin, riboflavin, calcium, magnesium, and selenium lower than the EAR [72]. Our estimate that nearly one third of U.S. adults is at risk of deficiency in at least one vitamin or has anemia is conservative and does not reflect the full scale of micronutrient deficiencies, as the number of micronutrients analyzed was limited to those with biochemical measures.

Despite evidence that micronutrient intake from the diet is generally higher in those using DS [29,62,63], 14% of FSMV users and 28% of DS users were at risk of deficiency in at least one vitamin or had anemia. Even when FSMV users were consuming a diet that met the EAR or RDA, 4.8% and 0.9% respectively were deficient in at least one vitamin or had anemia. We did not have access to vitamin D intake data; in fact, the dietary contribution of this micronutrient to nutrient status is difficult to determine as cutaneous synthesis is able to provide adequate vitamin D. Due to the way that we selected the FSMV, it is possible that some products do not meet the RDA for all of the vitamins and minerals, and this means that dietary gaps still exist despite FSMV use. The form of vitamin or mineral also affects absorption, which is not always taken into account when DS are being formulated. Nutrient–nutrient interactions can also mean that biochemical deficiency results from the low intake of a nutrient not captured by the NHANES dataset. For example, biochemical riboflavin status, which was not measured within NHANES 2003–2006, can affect iron homeostasis and anemia prevalence [67].

The most common deficiency was vitamin B6. The main symptoms of frank vitamin B6 deficiency include microcytic anemia, convulsions, depression, and confusion [7]. Marginal deficiency is associated with cardiovascular disease, and an elevated risk of Alzheimer’s disease [73]. There is a clear, positive correlation between vitamin B6 intake and status [49]. We assume that increasing vitamin B6 intake, particularly for women aged 19–50 years, could reduce the risk of deficiency. There are few studies investigating how to improve the intake of vitamin B6, which is found in all food groups but has few excellent sources, however it seems that increasing the consumption of high-fiber cereals has been able to increase vitamin B6 status [74,75]. Increasing the consumption of organ meats, potatoes and other starchy vegetables, non-citrus fruits, fish, and poultry may also improve vitamin B6 intake and lower the risk of deficiency [5]. Attention to anemia and one of its major causes, poor iron intakes, in adult women, and to anemia and vitamin B12 status in the elderly, could make a significant impact on overall biochemical deficiency rates in the U.S. population. The relatively high rate of vitamin C deficiency found in adult men is most likely related to poor fruit and vegetable intakes [4]. There appear to be gender-based differences in motivators for fruit and vegetable consumption [76,77,78]. Knowledge of barriers to fruit and vegetable consumption in men, which seem to be related to a lack of interest in a healthy lifestyle and difficulties in food preparation, could be used to design interventions targeting vitamin C intakes and status. Increasing fruit and vegetable consumption may also improve the status of other micronutrients, such as vitamins A and B6 [79]. More consideration should be paid to the risk of vitamin D deficiency in minority populations [80]. Educational interventions to increase vitamin D status may be only marginally effective; food fortification may be more appropriate [81,82]. Our analysis implies that the use of DS, particularly FSMV, is associated with a reduced risk of biochemical deficiency. The low cost of FSMV, typically a few cents per day for generic brands (survey conducted on 6 June 2017 at a major online retailer and a large U.S.-based pharmacy chain, assuming a 200-count adult FSMV), make them an attractive prospect to ensure dietary adequacy. However, our results are derived from observational research, therefore it is prudent to consume nutritionally-dense foods.

Our finding that nearly one third of the U.S. population is at risk of vitamin deficiency or anemia is a concern, especially since our estimates are likely conservative and do not capture deficiency in all of the essential micronutrients. Yet, the public health significance of this finding is uncertain given that there is no guidance from national or international health organizations regarding acceptable levels of multiple, concurrent deficiencies in populations or their health significance. The identification of risk groups for deficiency such as adult women, non-Hispanic Blacks, and people of lower socio-economic status can help clinicians, dietitians, and public health professionals involved in nutrition interventions to identify deficiencies and tailor nutrition screening and prevention programs to be most effective.

## 5. Conclusions

The risk of vitamin deficiency or anemia is common in the U.S., and vulnerable groups include women, particularly those of child-bearing age, non-Hispanic Blacks, people of low socio-economic status, underweight and obese individuals, and individuals with poor diets. Adequate dietary intakes and the use of DS, particularly FSMV, are associated with a lower risk of deficiency. Nutrition intervention programs should use an approach targeted at vulnerable groups to reduce the overall burden of poor micronutrient status.

## Figures and Tables

**Table 1:** Deficiency risk criteria and risk of deficiency in individual vitamins or anemia.

Nutritional Biomarker	Deficiency Risk Criteria	Proportion Biochemically Deficient 2003–2004		Proportion Biochemically Deficient 2005–2006		Proportion Biochemically Deficient 2003–2006	
		%	SE	%	SE	%	SE
Vitamin A	Serum retinol <20 µg/dL [15]	0.28	0.65	0.25	0.84	0.26	0.05
Vitamin B6	PLP <20 nmol/L [15]	20 *	1.4	11 *	0.76	16	0.87
Vitamin B12	Serum vitamin B12 <200 pg/mL or MMA >0.271 µmol/L [15]	7.5 *	0.70	2.6 *	0.30	5.0	0.44
Folate	Red blood cell folate <95 ng/mL or serum folate <2 ng/mL [15]	0.37	0.10	0.18	0.40	0.27	0.05
Vitamin C	Serum ascorbic acid <0.2 mg/dL [15]	7.5	0.99	4.9	0.59	6.2	0.59
Vitamin D	25-hydroxyvitamin D <12 ng/mL [15]	7.9	1.2	9.8	1.2	8.9	0.83
Vitamin E	Alpha-tocopherol <500 µg/dL [15]	0.75	0.15	0.66	0.09	0.70	0.08
Anemia	Hemoglobin <13 g/dL (men ≥15 years) or <12 (women ≥15 years, adolescents 12–14 years) or <11 g/dL (pregnant women) or <11.5 g/dL (children >12); and mean cell volume <95 fL [40,41]	3.9	0.41	4.6	0.37	4.3	0.28

Data are from NHANES 2003–2006 representative of the U.S. population, aged ≥9 years, based on biochemical indicators of nutrient deficiency. Abbreviations: PLP, pyridoxal-5′-phosphate; MMA, methylmalonic acid; SE, standard error. * Cycles (2003–2004 or 2005–2006) differ significantly *p* < 0.05.

**Table 2:** Risk of multiple vitamin deficiencies and/or anemia according to demographic characteristics.

Characteristic	*N*	Deficient in 1 *		Deficient in 2 *		Deficient in 3–5 *		Not Deficient	
		%	SE	%	SE	%	SE	%	SE
All participants	13,225	23	0.78	6.3	0.49	1.7	0.18	69	1.2
Cycle									
2003–2004	6600	27	0.82 ^a^	7.2	0.64	1.9	0.29	63	1.4 ^a^
2005–2006	6625	19	1.1 ^b^	5.5	0.68	1.5	0.19	75	1.7 ^b^
Sex									
Male	6506	19 ^a^	1.1	4.6 ^a^	0.39	1.1	0.20	75 ^a^	1.4
Female	6719	26 ^b^	0.75	8.0 ^b^	0.64	2.3	0.29	64 ^b^	1.3
Ethnicity ^†^									
Mexican American	3195	24 ^a^	1.4	5.4 ^a^	0.61	1.2 ^a^	0.30	69 ^a^	2.1
Non-Hispanic White	5647	20 ^a^	1.1	5.4 ^a^	0.46	1.3 ^a^	0.19	73 ^a^	1.4
Non-Hispanic Black	3432	36 ^b^	1.2	14 ^b^	0.81	5.1 ^b^	0.37	45 ^b^	1.7
PIR (%)									
Low PIR, ≤1.85	5804	27 ^a^	0.92	9.8 ^a^	0.77	2.8 ^a^	0.27	60 ^a^	1.4
Medium PIR, >1.85 and ≤3.5	3224	24 ^a^	0.91	6.1 ^a^	0.80	1.8 ^ab^	0.26	68 ^b^	1.3
High PIR, >3.5	3570	18 ^b^	0.93	3.8 ^b^	0.43	0.82 ^b^	0.17	77 ^c^	1.1
Education ^‡^									
Less than high school	2433	27 ^a^	1.1	11 ^a^	0.84	2.9	0.43	59 ^a^	1.6
High school graduate	2105	26 ^ab^	1.2	8.0 ^ab^	0.91	2.2	0.44	64 ^a^	1.5
Some college/college graduate	4043	21 ^b^	1.0	5.0 ^b^	0.47	1.5	0.23	72 ^b^	1.3
BMI ^§^									
Underweight	280	23 ^ab^	2.6	10	2.4	8.4	1.7	58 ^ac^	4.1
Normal weight	2473	23 ^ab^	1.2	5.7	0.64	1.5 ^ab^	0.25	70 ^ab^	1.6
Overweight	2949	21 ^a^	0.90	5.4	0.51	0.98 ^a^	0.19	73 ^b^	1.1
Obese	2891	27 ^b^	1.3	9.4	0.88	2.8 ^b^	0.33	61 ^c^	1.8
Pregnancy status ^|^									
Positive	574	33	3.2	14	2.7	4.9	1.3	48	4.8
Negative	4520	27	0.88	7.5	0.72	2.1	0.34	63	1.3
Breastfeeding status ^¶^									
Breastfeeding a child	100	21	6.4 **	9.2	3.3 **	3.5	3.3 **	66	8.5
Not breastfeeding	269	35	4.7	15	3.2	3.9	1.6 **	47	4.9

The overall risk of vitamin deficiency or anemia was investigated according to age, gender, and life stage groups in Table 3. A risk of deficiency/anemia was most frequent in pregnant or breastfeeding women. Data are from NHANES 2003–2006 representative of the U.S. population, aged ≥9 years, based on biochemical indicators of nutrient deficiency. Abbreviations: PIR, poverty income ratio. * Risk of deficiency based on vitamins A, B6, B12, C, D, E, folate, or anemia. Different superscripts represent significant differences within demographical categories, *p* = 0.0125 using, for simplicity, the Bonferroni correction for five comparisons (the maximum number of sub-groups in the demographics categories included) and alpha = 0.05 for the entire table. ^†^ ”Other Hispanic” and “Other race” ethnicity categories not reported due to small sample size. ^‡^ Education status is restricted to adults aged 20 years and older. ^§^ Body mass index (BMI) categories are restricted to adults aged 20 years and older. ^|^ Percentages reflect proportion of women of childbearing potential: menstruating girls aged 8–11 and all women aged 12–59 years. ^¶^ Percentages reflect proportion of women 0 or 1 years postpartum at the time of the interview. ** Relative standard error >30%.

**Table 3:** Age, gender, and life stage categories and risk of deficiency.

Age, Gender and Life Stage Category	*N*	Deficient in 1 *		Deficient in 2 *		Deficient in 3–5 *		Not deficient	
		%	SE	%	SE	%	SE	%	SE
9–13 years, male & female	1734	15 ^b^	1.4	1.5 ^ab^	0.36	0.06 ^a^**^‡^	0.04	83 ^ab^	1.6
14–18 years, male	1242	18 ^b^	1.7	2.2 ^a^	0.65	0.13 ^ab^**^‡^	0.08	80 ^a^	1.9
14–18 years, female	1107	26 ^ab^	1.9	5.3 ^abc^	0.89	0.98 ^abcd^	0.23	68 ^bcd^	2.2
19–50 years, male	2442	20 ^b^	1.3	3.9 ^ab^	0.36	0.70 ^abc^	0.23	76 ^ab^	1.5
19–50 years, female	2150	30 ^a^	1.0	8.5 ^cd^	0.89	2.5 ^d^	0.43	59 ^d^	1.6
51–70 years, male & female	2347	21 ^b^	1.2	7.4 ^bcd^	0.90	2.0 ^cd^	0.27	70 ^abc^	1.7
71+ years, male & female	1540	23 ^b^	1.0	9.5 ^cd^	0.93	3.4 ^d^	0.48	64 ^cd^	1.7
Pregnant or breastfeeding	683	30 ^ab^	3.3	13 ^d^	2.2	4.6 ^d^	1.5	53 ^d^	4.3

Data are from NHANES 2003–2006 representative of the U.S. population, aged ≥9 years, based on biochemical indicators of nutrient deficiency. * Risk of deficiency based on vitamins A, B6, B12, C, D, E, folate, or anemia. Different superscripts represent significant differences between life stage categories, *p* < 0.00625 using Bonferroni correction for eight sub-groups and alpha = 0.05. ** Relative standard error >30%. ^‡^ Comparison made using Clopper–Pearson (exact) confidence interval.

**Table 4:** Percentage at risk of deficiency of individual vitamins, or anemia, by age, gender, and life stage categories.

Age, Gender, and Life Stage Category	Serum Retinol <20 µg/dL	PLP <20 nmol/L	Vitamin B12 <200 pg/mL or MMA >0.271 µmol/L	Serum Folate <2 ng/mL or RBC Folate <95 ng/mL	Vitamin C <0.2 mg/dL	Vitamin D <12 ng/mL	Vitamin E <500 µg/dL	Anemia and MCV <95 fL
9–13 years, male & female	0.41 (0.083, 1.2) *^‡^	9.4 (5.8, 12.9)	1.3 (0.30, 3.5) *^‡^	0.21 (0.011, 1.0) *^‡^	1.1 (0.33, 2.6) *^‡^	4.0 (2.2, 5.8)	1.4 (0.3, 2.5)	1.3 (0.5, 2.2)
14–18 years, male	0.034 (0, 0.53) *^‡^	5.6 (2.7, 8.4)	2.6 (0.6, 4.5)	0.20 (0.015, 0.85) *^‡^	3.2 (0.5, 5.8)	7.1 (3.9, 10.3)	3.7 (1.0, 6.3)	0.28 (0.034, 0.97) *^‡^
14–18 years, female	0.062 (0, 0.64) *^‡^	16 (11.2, 21.6)	2.2 (0.5, 4.0)	0.33 (0.047, 1.1) *^‡^	3.6 (0.7, 6.5)	10.6 (5.6, 15.6)	1.3 (0.1, 2.5) *	4.5 (2.2, 6.8)
19–50 years, male	0.20 (0.04, 0.61) *^‡^	8.1 (5.5, 10.6)	3.0 (1.9, 4.1)	0.25 (0.062, 0.67) *^‡^	8.7 (5.4, 11.9)	8.0 (5.2, 10.8)	0.5 (0.1, 1.0) *	0.9 (0.4, 1.5)
19–50 years, female	0.28 (0.04, 0.92) *^‡^	25 (20.3, 29.0)	3.9 (2.4, 5.4)	0.35 (0.074, 0.99) *^‡^	6.9 (4.3, 9.5)	12 (8.3, 15.3)	0.5 (0.1, 0.8)	6.6 (4.9, 8.3)
51–70 years, male & female	0.19 (0.018, 0.75) *^‡^	16 (12.6, 19.7)	6.9 (3.7, 10.1)	0.32 (0.076, 0.85) *^‡^	6.6 (4.0, 9.2)	8.4 (5.3, 11.5)	0.4 (0.1, 0.6)	4.0 (2.7, 5.3)
71+ years, male & female	0.27 (0.025, 1.0) *^‡^	15 (11.4, 18.8)	15 (10.5, 18.7)	0.10 (0.0020, 0.57) *^‡^	4.3 (2.3, 6.3)	9.1 (6.7, 11.5)	0.28 (0.047, 0.88) *^‡^	8.9 (6.2, 11.6)
Pregnant or breastfeeding	1.7 (0.12, 7.0) *^‡^	35 (25.4, 44.8)	4.5 (2.6, 6.3)	0.21 (0, 1.8) *^‡^	0.46 (0.047, 1.7) *^‡^	7.3 (2.0, 12.7)	2.0 (0.12, 8.5) *^‡^	18 (9.9, 25.3)

Data are from NHANES 2003–2006 representative of the U.S. population, aged ≥9 years, based on biochemical indicators of nutrient deficiency. Values represent percentage at risk of deficiency (99.375% confidence interval). Abbreviations: PLP, pyridoxal-5′-phosphate; MMA, methylmalonic acid; RBC, red blood cell; MCV, mean cell volume. * Relative standard error >30%; ^‡^ Clopper–Pearson (exact) confidence interval.

**Table 5:** Risk of vitamin deficiency or anemia by type of DS reportedly used by survey respondents, according to age, gender and life stage category.

	Not Taking a DS				Taking a DS but Not an FSMV				Taking an FSMV			
	*N*	% DS Use *	% Deficient ^†^	SE	*N*	% DS Use *	% Deficient ^†^	SE	*N*	% DS Use *	% Deficient ^†^	SE
Entire Dataset	7281	44	40 ^a^	1.3	4312	40	28 ^b^	1.5	1615	16	14 ^c^	0.9
Age, gender and life stage category												
9–13 years, male & female	1321	68	20	1.7	260	20	10	2.5	150	12	18	4.5
14–18 years, male	1007	75	23	2.1	182	20	14	3.9	52	4.8	12	6.0
14–18 years, female	861	69	37 ^a^	2.8	194	25	25 ^ab^	3.7	50	6.2	13 ^b^	6.2
19–50 years, male	1469	52	33 ^a^	1.8	740	36	17 ^b^	2.1	232	12	7.9 ^b^	1.7
19–50 years, female	1138	43	54 ^a^	2.0	828	47	35 ^b^	2.0	182	10	19 ^b^	2.9
51–70 years, male & female	869	30	47 ^a^	3.1	957	44	29 ^b^	2.0	515	26	13 ^c^	1.5
71+ years, male & female	459	25	58 ^a^	3.5	731	49	34 ^b^	2.2	348	26	17 ^c^	2.5
Pregnant or breastfeeding	157	26	55	7.3	420	63	46	4.5	86	11	34	7.4

Data are from NHANES 2003–2006 representative of the U.S. population, aged ≥9 years, based on biochemical indicators of nutrient deficiency. Abbreviations: DS, dietary supplement; FSMV, full-spectrum multivitamin-multimineral supplement containing 12 or more vitamins and 7 to 16 minerals. * Percentage of participants in indicated DS use category, representative of the U.S. population, aged ≥9 years. † Deficiency prevalence, based on vitamins A, B6, B12, C, D, E, folate, or anemia. Different superscripts represent significant differences between DS categories within one category of inadequate status, *p* < 0.0167 using Bonferroni correction for three comparisons and alpha = 0.05.

## Supplementary Materials

The following are available online at http://www.mdpi.com/2072-6643/9/7/655/s1, Table S1: Proportion of population with Usual Intakes below the EAR, RDA and above the TUL for 6 vitamins and iron in the U.S. population >9 y, from NHANES 2003–2006, Table S2: Dietary inadequacy score in the U.S. population aged ≥9 y, based on NHANES 2003–2006, Table S3: Total counts of vitamins and minerals in dietary supplement products used in NHANES 2003–2006, Table S4: Demographic characteristics in the U.S. population aged ≥9 y from NHANES, 2003–2006, Table S5: Biochemical markers of nutrient status in the U.S. population, aged ≥9 y from NHANES, 2003–2006, Table S6: Risk of deficiency and length of time taking dietary supplements, Table S7: Risk of deficiency and frequency taking dietary supplements over previous 30 days.
