# Supplementary material for: Risk of Deficiency in Multiple Concurrent Micronutrients in Children and Adults in the United States
Source: Nutrients. 2017 Jun 24;9(7):655. doi: 10.3390/nu9070655 (PMC5537775; doi:10.3390/nu9070655)
Supplement: Supplementary file 1 [file nutrients-09-00655-s001.pdf]

Supplementary table S1: Proportion of population with Usual Intakes below the EAR, RDA and above the TUL for 6 vitamins and iron in the U.S. population > 9 y, from NHANES 2003-2006

Vitamin or mineral	N	Proportion with inadequate intake according to EAR[4]		Proportion with insufficient intake according to RDA[4]		Proportion with excessive intake according to TUL[4]	
		%	SE	%	SE	%	SE
Vitamin A[1]	13027	51.5	1.0	72.1	0.9	0.31	0.01
Vitamin B6	13027	21.2	0.9	32.0	1.0	0	-
Vitamin B12[2]	13027	12.2	0.6	18.1	0.7	-	-
Folate[3]	13027	21.2	0.8	35.3	0.9	0.68	0.12
Vitamin C	13027	48.0	1.0	56.1	1.1	0	-
Vitamin E	13027	88.9	0.5	94.4	0.4	0	-
Iron	13027	7.3	0.4	29.3	0.9	1.1	0.13

EAR, Estimated Average Requirement; NHANES, National Health and Nutrition Examination Survey; RDA, Recommended Dietary Allowance; TUL, Tolerable Upper Limit.

[1]For calculating the proportion meeting the EAR and RDA for vitamin A, total intake is based on Retinol Activity Equivalents (based on intake of pre-formed vitamin A as retinol, and intake of pro-vitamin A carotenoids), while retinol intakes are used to calculate the TUL (from: Otten, J. J., et al., Eds. (2006). Dietary reference intakes: the essential guide to nutrient requirements. Washington, DC., The National Academies Press.)

[2]No TUL is defined for vitamin B12

[3]For calculating the proportion meeting the EAR and RDA for folate, total intake was based on naturally occurring folates in foods and folic acid in fortified foods and dietary supplements, while only folate intakes from fortified foods and dietary supplements is used to calculate the TUL (from: Otten, J. J., et al., Eds. (2006). Dietary reference intakes: the essential guide to nutrient requirements. Washington, DC., The National Academies Press.)

[4]Values are are percentage of U.S. population aged ≥9 y not meeting the EAR or RDA, or exceeding the TUL, for individual age, gender and lifespan groups.

Supplementary table S2: Dietary inadequacy/insufficiency score in the U.S. population aged ≥9 y, based on NHANES 2003-2006

Dietary inadequacy/ insufficiency score*	Percentage of U.S. population, based on EAR			Percentage of U.S. population, based on RDA		
	N	%	SE	N	%	SE
0	667	6.4	0.34	228	2.3	0.25
1	3123	24	0.74	1603	12	0.49
2	3518	27	0.61	2901	21	0.63
3	2463	19	0.49	2719	21	0.55
4	1506	11	0.62	1898	15	0.42
5	891	6.3	0.39	1648	12	0.57
6	576	4.3	0.43	1227	8.9	0.49
7	283	1.9	0.15	803	6.1	0.40

EAR, Estimated Average Requirement; NHANES, National Health and Nutrition Examination Survey; RDA, Recommended Dietary Allowance; TUL, Tolerable Upper Limit.

*The dietary inadequacy/insufficiency score is based on not meeting the EAR or RDA for vitamins A, B6, B12, C, E, folate, or the mineral iron, for cut-off points based on participants' age, gender and pregnancy or breastfeeding status. N reflects crude counts. Percentages are weighted to be representative of the U.S. population.

Supplementary table S3: Total counts of vitamins and minerals in dietary supplement products used in NHANES 2003-2006

		Count of minerals in supplement																	
Count of vitamins in supplement		0	1	2	3	4	5	6	7	8	9	10	11	12	13	14	15	16	17+	*Total count*
	0	2941	710	308	80	19	7	3	1	2	3	6	3	5	2	1	0	0	12	*4103*
	1	902	379	123	45	9	5	25	3	4	1	2	3	2	0	0	0	0	0	*1503*
	2	184	65	58	22	7	8	11	6	2	1	0	2	0	0	0	0	0	0	*366*
	3	85	73	46	44	23	7	9	3	0	0	0	0	0	0	0	0	0	0	*290*
	4	28	13	17	17	11	7	2	7	0	0	0	0	2	0	0	0	0	0	*104*
	5	19	13	10	9	6	10	0	2	4	0	1	0	0	0	0	0	0	0	*74*
	6	23	18	11	9	1	4	2	5	0	0	0	0	0	0	0	0	0	0	*73*
	7	18	9	4	9	10	3	0	2	0	0	0	0	0	0	0	0	0	0	*55*
	8	65	33	19	5	5	17	3	2	0	0	0	0	0	0	0	0	0	0	*149*
	9	135	35	6	21	12	14	5	4	0	0	1	0	1	0	0	0	0	1	*235*
	10	91	78	42	75	51	40	7	9	4	2	0	1	2	0	0	0	0	0	*402*
	11	55	31	7	27	27	5	11	12	18	4	6	1	5	0	0	0	0	0	*209*
	12	13	15	7	5	9	20	21	**68**	**49**	**23**	**33**	**47**	**11**	**4**	**6**	**2**	**4**	1	*338*
	13	1	7	2	3	1	6	9	**74**	**75**	**89**	**93**	**93**	**33**	**34**	**95**	**67**	**202**	5	*889*
	14	3	1	0	0	0	0	2	**3**	**9**	**20**	**40**	**49**	**59**	**26**	**6**	**0**	**5**	3	*226*
	16	0	0	0	0	0	0	0	**0**	**0**	**0**	**2**	**3**	**0**	**0**	**0**	**0**	**0**	0	*5*
	17	0	0	0	0	0	0	0	**0**	**0**	**0**	**1**	**0**	**0**	**0**	**0**	**0**	**0**	0	*1*
	18	0	0	0	0	0	0	0	**0**	**0**	**0**	**0**	**1**	**1**	**1**	**0**	**0**	**0**	0	*3*
	Total count	*4564*	*1480*	*660*	*371*	*191*	*153*	*110*	*201*	*167*	*146*	*188*	*204*	*131*	*68*	*108*	*69*	*211*	*22*	*9044*

NHANES, National Health and Nutrition Examination Survey.
Bold = Full Spectrum Multivitamin-Multimineral category (FSMV), green text = 50+ products, yellow text = 100-200 products, red text = 200+ products

Supplementary table S4: Demographic characteristics in the U.S. population aged ≥9 y from NHANES, 2003-2006

Characteristic	*N*	Value (%)	
		%	SE
Sex			
Male	6506	48.9	0.4
Female	6719	51.1	0.4
Age category			
9-13 years	1734	7.0	0.27
14-18 years	2424	8.0	0.31
19-50 years	5180	53	1.0
51-70 years	2347	23	0.86
71 years and over	1540	9.2	0.58
Ethnicity			
Non-Hispanic White	5647	70.6	2.3
Non-Hispanic Black	3432	11.8	1.4
Mexican American	3195	8.48	1.1
Other Hispanic	399	3.65	0.5
Other race	552	5.38	0.5
PIR			
Low PIR, =< 1.85	5804	31.0	1.4
Medium PIR, >1.85-<=3.5	3224	27.7	1.0
High PIR, >3.5	3570	41.3	1.6
Education[1]			
Less than high school	2433	17.7	0.9
High school graduate	2105	26.0	0.7
Some college, or college graduate	4043	56.3	1.1
BMI[2]			
Underweight	280	3.03	0.21
Normal weight	2473	30.9	0.85
Overweight	2949	33.3	0.70
Obese	2891	32.7	0.92
DS user			
No use	7281	44.3	0.87
FSMV use	4312	15.5	0.60
Any other DS	1615	40.2	0.65
Pregnancy status[3]			
Positive	574	4.13	0.27
Negative	4520	95.9	0.27
Breastfeeding status[4]			
Breastfeeding a child	100	27.8	4.6
Not breastfeeding	269	72.1	4.6

BMI, Body Mass Index; NHANES, National Health and Nutrition Examination Survey; PIR, poverty income ratio; FSMV, full spectrum multivitamin-multimineral; DS, Dietary supplement.

[1]Education status is restricted to adults aged 20 y and older. [2]BMI categories are restricted to adults aged 20 y and older. [3]Percentages reflect proportion of women of childbearing potential, aged 9 to 59 y. [4]Percentages reflect proportion of women 0 or 1 y postpartum at the time of the interview.

Supplementary table S5: Biochemical markers of nutrient status in the U.S. population, aged ≥9 y from NHANES, 2003-2006

**Parameter**	***N***	**2003-2004**		**2005-2006**	
		Geometric mean	95% CI	Geometric mean	95% CI
**Retinol, µg/dL**	13740	55.0	54.5-55.5	55.5	55.0-56.1
**Serum folate, ng/mL**	13891	11.8	11.6-12.0	12.1	11.9-12.3
**RBC folate, ng/mL**	13963	258	255-261	271	268-275
**Vitamin B6 (PLP), nmol/L**	13831	42.6	41.3-43.9	50.3	49.0-51.7
**Serum vitamin B12, pg/mL**	13815	472	465-478	485	477-492
**MMA, µmol/L**	6741	0.135	0.133-0.137	-	
**Vitamin C, mg/dL**	13778	0.779	0.760-0.799	0.819	0.801-0.837
**Vitamin D, ng/mL**	13969	22.0	21.8-22.3	21.5	21.3-21.8
**Vitamin E, µg/dL alpha-tocopherol**	13740	1173	1157-1189	1101	1089-1114
		Arithmetic mean		Arithmetic mean	
**Haemoglobin, g/dL**	14053	14.4	14.4-14.5	14.4	14.3-14.5
**Mean cell volume, fL**	14053	89.9	89.7-90.0	89.5	89.5-90.2

NHANES, National Health and Nutrition Examination Survey; RBC, red blood cell; PLP, pyridoxal-5'-phosphate; MMA, methylmalonic acid.

Supplementary table S6: Risk of deficiency and length of time taking dietary supplements

Length of time using dietary supplement	Less than 2 months		2 months to 6 months		Greater than 6 months	
N	949		839		5058	
Dietary supplement users						
	%	SE	%	SE	%	SE
Not deficient	67.1	1.9	71.1	2.86	73.8	1.7
Deficient in 1 or more vitamins or minerals	32.9	1.9	28.9	2.86	26.3	1.7
FSMV users						
Not deficient	82.7	2.9	90.5	2.4	86.4	1.0
Deficient in 1 or more vitamins or minerals	17.3	2.9	9.54	2.4	13.6	1.0

FSMV, full spectrum multivitamin-multimineral.

Supplementary table S7: Risk of deficiency and frequency taking dietary supplements over previous 30 days

Frequency taking dietary supplement in previous 30 days	Less than four times		Four to 15 times		16 to 29 times		30 times	
N	1316		1351		762		3296	
Dietary supplement users								
	%	SE	%	SE	%	SE	%	SE
Not deficient	68.1	2.1	70.5	2.5	76.4	2.7	76.0	1.3
Deficient in 1 or more vitamins or minerals	31.9	2.1	29.5	2.5	23.6	2.7	24.0	1.3
FSMV users								
Not deficient	83.1	2.9	82.7	3.3	88.9	2.6	87.9	0.89
Deficient in 1 or more vitamins or minerals	16.9	2.9	17.3	3.3	11.1	2.6	12.1	0.89

FSMV, full spectrum multivitamin-multimineral.
